# Supplemental bifid triple viable capsule treatment improves inflammatory response and T cell frequency in ulcerative colitis patients

**DOI:** 10.1186/s12876-021-01887-2

**Published:** 2021-08-04

**Authors:** Shuying Li, Yan Yin, Dan Xiao, Yong Zou

**Affiliations:** 1 General Department, The Sixth Hospital of Wuhan, Affiliated Hospital of Jianghan University, Wuhan, China; 2 Department of Infection Management, The Sixth Hospital of Wuhan, Affiliated Hospital of Jianghan University, Wuhan, China; 3 Department of Gastroenterology, The Sixth Hospital of Wuhan, Affiliated Hospital of Jianghan University, No.168 Hongkong Road, Wuhan, 430015 China; 4 Emergency Department, Wuhan Emergency Medical Center, No.288 Machang Road, Jianghan District, Wuhan, 430022 China

**Keywords:** Mesalazine, Somatostatin, Bifid triple viable capsules, Ulcerative colitis, Plasma inflammatory factors, T cell frequency

## Abstract

**Background:**

Ulcerative colitis is a common non-specific chronic disease. Supplementing probiotics has become an important method for the treatment of ulcerative colitis. This study aimed to explore the effect of supplementing bifid triple viable capsules on background mesalazine plus somatostatin on plasma inflammatory factors and T cell frequency in ulcerative colitis patients.

**Methods:**

A total of 130 ulcerative colitis patients admitted to our hospital from August 2018 to March 2020 were included and divided into the experimental group (65 patients with mesalazine plus somatostatin and bifid triple viable capsules for treatment) and the control group (65 patients treated with mesalazine plus somatostatin) using the random number table method. Bifid triple viable bacteria capsules were given orally, 420 mg each time, with 3 times a day for 2 months.

**Results:**

Before treatment, the plasma levels of IL-6, IL-8, hs-CRP, TNF-α, D-lactic acid, and endotoxin (ET), CD4+, CD8+, CD4/CD8 ratio, diamine oxidase (DA0), emotional ability, social ability, intestinal and systemic symptoms were not significantly different between the two groups (all *P* > 0.05). After treatment, the plasma levels of IL-6, IL-8, hs-CRP, and TNF-α decreased in both groups, and were lower in the experimental group than those in the control group (all *P* < 0.05). The levels of CD4+ and CD4/CD8 ratio increased, and were higher in the experimental group than those in the control group (*P* < 0.05); the CD8+ levels were reduced, and were lower in the experimental group than those in the control group (*P* < 0.05). The plasma D-lactic acid, ET, and DA0 levels were decreased, and were lower in the experimental group than those in the control group; emotional ability, social ability, intestinal and systemic symptoms were improved, and were higher in the experimental group than those in the control group (all *P* < 0.05). During the course of treatment, 2 cases of abdominal discomfort and 1 case of rash occurred in the experimental group, with an adverse event rate of 4.62% (3/65); 3 cases of abdominal discomfort and 2 cases of rash occurred in the control group, with an adverse event rate of 7.69% (5/65).

**Conclusion:**

The supplementary treatment of bifid triple viable capsules can effectively enhance the curative effect in ulcerative colitis patients, reduce plasma inflammatory factors, and regulate T cell frequency, which is worthy of clinical application.

## Background

Ulcerative colitis is a common non-specific chronic disease, which can involve the large intestine mucosa and submucosa, with abdominal pain, mucus pus and blood in the stool, and diarrhea as the main clinical manifestations [1]. It has a protracted course, is extremely difficult to treat and recurs easily, which has a serious impact on the patient's normal life and work. Therefore, selecting an appropriate treatment regimen is a key clinical issue that needs to be resolved urgently. Recent studies have confirmed that the main characteristic of ulcerative colitis is that the intestinal wall is infiltrated and continuously activated by a large number of inflammatory cells, for which the occurrence and development are closely related to a variety of inflammatory factors such as interleukin-6 (IL-6), interleukin-8 (IL-8), high-sensitivity C-reactive protein (hs-CRP) and tumor necrosis factor (TNF)-α [2]. Currently, there are few clinically effective methods for the treatment of ulcerative colitis. Antibacterial agents, glucocorticoids, steroid hormones and aminosalicylic acid preparations are commonly used, in which the first choice is the aminosalicylic acid preparations [3].

With the deepening of research and the development of the medical levels, it has been found that ulcerative colitis is closely related to an imbalance of the intestinal flora [4]. The addition of probiotics during treatment has become a novel treatment concept and method [5]. Probiotics have a promoting effect on maintaining the balance of the flora in the intestinal tract and can be used for the treatment of a variety of intestinal diseases, and play a good auxiliary synergistic effect [6]. The composition and proportion of the intestinal flora play an important role in the occurrence and progression of ulcerative colitis [7]. When the intestinal flora is disturbed, the food residues in the intestine are abnormally fermented under the action of harmful bacteria to produce detrimental substances, causing damage to the intestinal cavity and mucous membranes. As a result, patients may develop abdominal pain, diarrhea, hematochezia and other symptoms, resulting in the occurrence of ulcerative colitis or aggravation of the disease [8]. Bifid triple viable capsules can promote local granulation growth and vascular remodeling of the intestinal ulcer tissues and accelerate rapid healing of the ulcer surface. In addition, bifid triple viable capsules can increase the content of probiotics, improve the barrier function and immune function of the intestinal tract, thereby reducing the inflammatory response. Research conducted by Zhang et al. [9, 10] confirmed that the bifid triple viable bacteria can produce lactic acid and acetic acid after entering the body, reduce the pH of the intestinal lesions, inhibit the reproduction of harmful bacteria, and increase the proportion of intestinal probiotics by adjusting the proportion of intestinal flora, and restore the normal balance of the intestinal flora of the body. Somatostatin is a peptide hormone with 14 amino acids isolated from the hypothalamus, which is a neuropeptide with a variety of physiological functions [11]. It can inhibit large amounts of vasodilators such as vasoactive intestinal peptides and the secretion of cellular inflammatory factors such as interleukins, reduce hepatic artery blood flow and intrahepatic vascular resistance, protect gastrointestinal mucosa, and relieve inflammation symptoms [10]. Somatostatin has a certain protective effect on ulcerative colitis patients, and it also has a variety of regulatory effects on the immune system [11]. Mesalazine has the function of inhibiting colonic mucosa secretion [12].

However, there are few clinical reports on the treatment of ulcerative colitis with mesalazine plus somatostatin and bifid triple viable capsules. Therefore, this study enrolled 130 ulcerative colitis patients admitted to our hospital and explored the effect of mesalazine plus somatostatin and bifid triple viable capsules on plasma inflammatory factors and T cell frequency in ulcerative colitis patients, thereby providing a basis for the treatment of ulcerative colitis.

## Methods

### Clinical data

A total of 130 ulcerative colitis patients who were admitted to our hospital from August 2018 to March 2020 were included and divided into the experimental group and the control group using the random number table method. There were 65 cases in each group. The study group were 27–55 years old, with a mean age of (40.83 ± 3.21) years, and had 33 males and 32 females; the control group were 28–54 years old, with a mean age of (40.79 ± 3.19) years, and had 34 males and 31 females. There was no statistically significant difference in general data between the two groups (*P* > 0.05, Table 1). All patients voluntarily participated in the study and signed an informed consent form, and the study protocol complied with the relevant requirements of the Declaration of Helsinki of the World Medical Association. This study was approved by the Ethics Committee of Affiliated Hospital of Jianghan University.

**Table 1:** General data of patients in the two groups

	Experimental group (n = 65)	Control group (n = 65)	χ^2^ vale	*P* value
Age (years)	40.83 ± 3.21	40.79 ± 3.19	0.071	0.944
Male/female	33/32	34/31	0.031	0.861
BMI (kg/m^2^)	23.67 ± 0.21	23.66 ± 0.19	0.285	0.776
Course of disease (h)	24.36 ± 2.08	24.21 ± 1.76	0.444	0.658
Degree of disease (n)				
Mild	19	21	0.144	0.704
Moderate	46	44		
Range of lesions (n)				
Left hemi-colon	17	15	0.166	0.684
Pancolitis	48	50		
Pre-treatment mayo score (points)				
Mild	7.21 ± 0.63	7.13 ± 0.59	0.415	0.680
Moderate	8.26 ± 2.29	8.58 ± 2.13	− 0.458	0.649

BMI, Body Mass Index

### Inclusion and exclusion criteria

The inclusion criteria were as follows: patients met the diagnostic criteria for ulcerative colitis in the "Interpretation of Guidelines for the Diagnosis and Drug Treatment of Inflammatory Bowel Disease" [13], with typical clinical symptoms, and were diagnosed as ulcerative colitis by fiber colonoscopy; patients with a clear history and allergy history, complete clinical data; patients had clear consciousness, and stable vital signs; patients with good compliance and normal mental health. Exclusion criteria were as follows: patients had serious primary diseases in systems such as the cardiovascular, liver, kidney and hematopoietic system; patients had organic diseases such as colon cancer, disease of the biliary tract and pancreas, etc.; patients had endocrine and metabolic diseases such as blood coagulation, hematologic diseases, and genetic diseases, etc.; patients had other gastrointestinal diseases (bacterial dysentery, Crohn's disease, and ischemic colitis, etc.); pregnant or lactating women; patients were allergic to the drugs in this study; patients received surgical treatment, radiotherapy and chemotherapy and other drug treatments; patients did not strictly follow the study protocol.

### Treatment regimen

All patients were given nutritional intervention, and were informed to eat more easily digestible foods, to ensure the intake of high-protein substances, and avpod spicy and stimulating foods. Patients in the control group received mesalazine plus somatostatin, in which mesalamine was taken orally [manufacturer: Sunflower Pharmaceutical Group Jiamusi Luling Pharmaceutical Co., Ltd.; Approval Number: National Medicine Standard H19980148], 1.0 g each time, 4 times a day; somatostatin [manufacturer: Swiss Serono Co., Ltd.; Approval Number: Registration Certificate No. X19990113] was dissolved in 5% glucose solution. It was first intravenously injected with 250 mg, and then immediately given intravenous administration at a rate of 250 mg·h- 1. Patients in the experimental group received bifid triple viable bacteria capsules in addition to the treatment in the control group. Bifid triple viable bacteria capsules [manufacturer: Shanghai Xinyi Pharmaceutical Co., Ltd.; approval number: National Medicine Standard S10950032] were given orally, 420 mg each time, 3 times a day. The above-mentioned treatments were continued for 2 months.

### Observational indicators

The clinical efficacy and adverse events (AEs) in the two groups of patients were observed after treatment, and the plasma inflammatory factors (IL-6, IL-8, hs-CRP, and TNF-α)] and T cell frequency (T lymphocyte subpopulations CD4  and CD8+, and CD4/CD8 ratio), markers for intestinal mucosal barrier function [plasma D-actic acid, plasma endotoxin (ET), and diamine oxidase (DA0)] and quality of life (QoL) before and after treatment were compared.

Clinical efficacy: according to the "Consensus Opinions on the Diagnostic and Treatment Standards of Inflammatory Bowel Disease in China" [14], the clinical efficacy was evaluated. Cure: clinical symptoms and signs disappeared; endoscopic colonoscopy showed no obvious abnormalities in the intestinal mucosa, and no recurrence during follow-up; markedly effective: clinical symptoms and signs largely disappeared, and colonoscopy showed mild mucosal inflammation; effective: clinical symptoms and signs were improved, and colonoscopy showed improvement in the intestinal mucosa; invalid: none of the above standards had changed. Total effective rate = effective rate + markedly effective rate + cure rate.

Laboratory examinations: 3–5 ml of cubital venous blood was drawn before and after treatment, and the plasma was split and stored in a refrigerator at -45℃ after centrifugation. IL-6, IL-8, hs-CRP, TNF-α, and ET were determined by enzyme-linked immunosorbent assay; CD4+, CD8+, and CD4/CD8 ratio were tested using a Beckman Coulter DxFLEX flow cytometer. The levels of plasma D-lactic acid and DA0 were detected by enzymatic spectrophotometry; all the kits were purchased from Shanghai Enzyme-Linked Biotechnology Co., Ltd. and operated in strict accordance with the instructions.

QoL: The Inflammatory Bowel Disease Questionnaire (IBDQ) [15] was used before and after treatment for evaluating the QoL in patients between the two groups. The scale included 4 dimensions: emotional ability, social ability, intestinal and systemic symptoms, with a total of 32 items. Each item had a score of 1–7, with a full score of 32–224. The higher the score was, the higher the QoL of the patient was.

AEs: AEs such as abdominal discomfort during the treatment were recorded in detail.

### Statistical analysis

The IBM Microsoft SPSS 21.0 software was used for statistical analysis of data. Measurement data was first tested for normality, and the data met the normal distribution was expressed as ${\overline{\text{x}}} \pm {\text{s}}$. Two independent sample t test was used for comparisons between groups, and paired t test was used for comparisons before and after treatment within the group; categorical data was expressed as rate (%), and Chi-square χ^2^ test was used for comparison. *P* < 0.05 was considered as statistically significant.

## Results

### Comparison of clinical efficacy between the two groups

In the experimental group, the number of cases with cure, markedly effective, effective and invalid treatment was 35, 17, 8, and 5, respectively, with an effective rate of 92.31%. The number of cases with cure, markedly effective, effective and invalid treatment in the control group was 21, 12, 17, and 15, respectively, with an effective rate of 76.92%. The difference in effective rate between the two groups was statistically significant (χ^2^ = 5.909, *P* = 0.015).

### Comparison of plasma inflammatory factors between the two groups

The levels of plasma inflammatory factors between the two groups were comparable before treatment (all *P* > 0.05). After treatment, the IL-6, IL-8, hs-CRP, and TNF-α of the two groups decreased, and were significantly lower in the experimental group than those in the control group (all *P* < 0.05, Table 2);

**Table 2:** Comparison of serum inflammatory factors between the two groups (${\overline{\text{x}}} \pm {\text{s}}$)

	Experimental group (n = 65)			Control group (n = 65)			t#	P# value	T$	P^$^ value
	Before treatment	After treatment	Difference between before and after treatment	Before treatment	After treatment	Difference between before and after treatment				
IL-6 (ng·L-1)	172.09 ± 15.29	90.01 ± 8.02*	82.08 ± 7.27	171.89 ± 16.21	101.27 ± 7.27*	70.62 ± 8.94	− 8.386	< 0.001	8.018	< 0.001
IL-8 (ng·L-1)	269.08 ± 12.09	152.63 ± 18.72*	116.45 ± 6.63	268.91 ± 11.78	160.92 ± 12.29*	107.99 ± 0.51	− 2.985	.003	10.257	< 0.001
Hs-CRP (ng·L-1)	74.82 ± 5.29	6.83 ± 1.01*	67.99 ± 4.28	74.21 ± 5.21	9.21 ± 0.76*	65.00 ± 4.45	− 15.180	< 0.001	3.904	< 0.001
TNF-α (ng·L-1)	108.93 ± 9.03	61.87 ± 6.38*	47.06 ± 2.65	108.27 ± 8.92	68.29 ± 7.11*	39.98 ± 1.81	− 5.418	< 0.001	17.787	< 0.001

IL, interleukin; Hs-CRP, high-sensitivity C-reactive protein; TNF, tumor necrosis factor

^*^Compared with that of before treatment, *P* < 0.001

^#^Comparisons between experimental group and control group after treatment

^$^Comparisons of difference in the values before and after treatment between the two groups

### Comparison of T cell frequency between the two groups

The levels of CD4+, CD8+, and CD4/CD8 were comparable between the two groups before treatment (all *P* > 0.05). The levels of CD4+ and CD4/CD8 ratio increased after treatment, and were significantly higher in the experimental group than those in the control group (*P* < 0.05, Table 3); the CD8+ levels were reduced, and were significantly lower in the experimental group than those in the control group (*P* < 0.05, Table 3);

**Table 3:** Comparison of immune functions between the two groups (${\overline{\text{x}}} \pm {\text{s}}$)

	Experimental group (n = 65)			Control group (n = 65)			t^#^	*P*^#^ value	t^$^	*P*^$^ value
	Before treatment	After treatment	Difference between before and after treatment	Before treatment	After treatment	Difference between before and after treatment				
CD4 + (%)	35.46 ± 3.29	46.37 ± 4.21*	10.91 ± 0.92	35.41 ± 3.07	40.02 ± 3.21*	4.61 ± 0.14	9.670	< 0.001	54.581	< 0.001
CD8 + (%)	33.47 ± 3.01	26.36 ± 2.99*	7.11 ± 0.02	33.52 ± 2.98	29.51 ± 2.73*	4.01 ± 0.25	− 6.272	< 0.001	99.654	< 0.001
CD4/CD8 ratio	1.03 ± 0.18	1.79 ± 0.14*	0.76 ± 0.04	1.05 ± 0.19	1.41 ± 0.09*	0.36 ± 0.10	18.408	< 0.001	29.942	< 0.001

CD, cluster of differentiation

^*^Compared with that of before treatment, *P* < 0.001

^#^Comparisons between experimental group and control group after treatment

^$^Comparisons of difference in the values before and after treatment between the two groups

### Comparisons of markers for intestinal mucosal barrier function between the two groups

There was no significant difference in the plasma D-lactic acid, ET, and DA0 levels between the two groups before treatment (all *P* > 0.05). After treatment, the plasma D-lactic acid, ET, and DA0 levels were decreased, and were lower in the experimental group than those in the control group (All *P* < 0.05, Table 4);

**Table 4:** Comparisons of intestinal mucosal barrier function between the two groups (${\overline{\text{x}}} \pm {\text{s}}$)

	Experimental group (n = 65)			Control group (n = 65)			t^#^	*P*^#^ value	t^$^	*P*^$^ value
	Before treatment	After treatment	Difference between before and after treatment	Before treatment	After treatment	Difference between before and after treatment				
Serum D lactic acid (mmol·L^−1^)	5.62 ± 0.39	4.01 ± 0.08*	1.61 ± 0.31	5.63 ± 0.38	4.61 ± 0.02*	5.25 ± 0.36	− 58.662	< 0.001	− 61.772	< 0.001
ET (Eu·mL^−1^)	0.25 ± 0.03	0.13 ± 0.02*	0.12 ± 0.01	0.24 ± 0.05	0.19 ± 0.01*	0.05 ± 0.04	− 21.633	< 0.001	13.688	< 0.001
DA0 (U·L^−1^)	8.01 ± 1.02	5.38 ± 0.32*	2.63 ± 0.70	7.99 ± 0.97	6.83 ± 0.65*	1.16 ± 0.32	− 16.136	< 0.001	15.398	< 0.001

ET, endotoxin; DA0, diamine oxidase

^*^Compared with that of before treatment, *P* < 0.001

^#^Comparisons between experimental group and control group after treatment

^$^Comparisons of difference in the values before and after treatment between the two groups

### Comparisons of QoL between the two groups

There was no significant difference in emotional ability, social ability, intestinal and systemic symptoms between the two groups before treatment (all *P* > 0.05). After treatment, emotional ability, social ability, intestinal and systemic symptoms were improved in the two groups, and the scores were higher in the experimental group than those in the control group (all *P* < 0.05, Table 5).

**Table 5:** Comparisons of quality of life between the two groups (${\overline{\text{x}}} \pm {\text{s}}$)

	Experimental group (n = 65)			Control group (n = 65)			t^#^	*P*^#^ value	t^$^	*P*^$^ value
	Before treatment	After treatment	Difference between before and after treatment	Before treatment	After treatment	Difference between before and after treatment				
Emotional ability	32.81 ± 3.23	48.93 ± 4.01*	16.12 ± 0.78	32.78 ± 3.19	42.37 ± 3.27*	9.59 ± 0.08	10.221	< 0.001	67.143	< 0.001
Social ability	30.92 ± 3.28	50.93 ± 6.01*	20.01 ± 2.73	30.87 ± 3.18	45.88 ± 4.23*	15.01 ± 1.05	5.54	< 0.001	13.782	< 0.001
Intestinal symptoms	25.03 ± 2.14	45.39 ± 3.01*	20.36 ± 0.87	24.96 ± 2.03	30.61 ± 3.21*	5.65 ± 1.18	27.079	< 0.001	80.895	< 0.001
Systemic symptoms	38.92 ± 3.12	55.83 ± 4.82	16.91 ± 1.70	38.89 ± 3.09	48.93 ± 4.29	10.04 ± 1.20	8.621	< 0.001	26.618	< 0.001

^*^Compared with that of before treatment, *P* < 0.001

^#^Comparisons between experimental group and control group after treatment

^$^Comparisons of difference in the values before and after treatment between the two groups

### Comparisons of AEs between the two groups

During treatment, 2 cases of abdominal discomfort and 1 case of rash occurred in the experimental group, with an AE rate of 4.62% (3/65); 3 cases of abdominal discomfort and 2 cases of rash occurred in the control group, with an AE rate of 7.69% (5/65); there was no significant difference in the occurrence rate of AEs between the two groups (χ^2^ = 0.533, *P* = 0.718).

## Discussion

Bifid triple viable capsule is a kind of probiotics composed of enterococcus, lactobacillus acidophilus and probiotic bifidobacterium, which can supplement the original intestinal flora, inhibit pathogenic bacteria, adhesion and forming a with intestinal mucosal epithelial cells. It has a protective effect on the intestinal mucosa, can prevent the invasion and colonization of pathogenic bacteria and opportunistic pathogens, regulate the imbalance of the intestinal flora, and improve clinical symptoms. Song Taoyan et al. [12] confirmed that mesalamine plus bifid triple viable capsules can effectively improve the clinical symptoms of ulcerative colitis patients. Zhang Jing et al. [10] showed that somatostatin plus mesalazine can significantly improve the intestinal function of ulcerative colitis patients. The results of this study showed that the clinical efficacy of the experimental group was higher than that of the control group (*P* < 0.05), suggesting that mesalamine plus somatostatin and bifid triple viable capsules can effectively improve the clinical symptoms of ulcerative colitis patients.

Inflammatory cytokines play an important role in the occurrence and development of ulcerative colitis [15]. The imbalance between anti-inflammatory and pro-inflammatory factors will accelerate the occurrence and progression of intestinal mucosal inflammation and is conducive to chronic development of inflammation [16]. IL-6, IL-8, hs-CRP, and TNF-α are all pro-inflammatory factors. IL-6 plays an important role in the occurrence and development of ulcerative colitis, and its expression can reflect the degree of inflammation of ulcerative colitis [17]. IL-8 mediates pathological damage of colon mucosa and induces the inflammatory response of the intestinal mucosa of ulcerative colitis, which plays an important role in the pathogenesis of ulcerative colitis [18]. It can be used as an important indicator to evaluate the severity of ulcerative colitis [18]. Hs-CRP can reflect the severity and activity of ulcerative colitis patients [19]. TNF-α can promote the chemotaxis of chemoattract neutrophils, causing tissue cell infiltration, and inflammatory damage of the intestinal mucosa [20]. Therefore, this study evaluated plasma inflammatory factors in ulcerative colitis patients treated with mesalazine plus somatostatin and bifid triple viable capsules. The results showed that IL-6, IL-8, hs- CRP and TNF-α were lower in the experimental group than those in the control group (*P* < 0.05), suggesting that mesalazine plus somatostatin and bifid triple viable capsules can effectively reduce the plasma inflammatory factor levels in ulcerative colitis patients. Jiang Shengjun [21] confirmed that mesalazine and bifid triple viable capsules plus compound glutamine enteric-coated capsules can improve the clinical symptoms of patients with mild to moderate ulcerative colitis during the active phase and reduce plasma inflammatory factors. Tan Yu'e [22] and colleagues have confirmed that the use of bifidobacteria and lactobacillus triple viable tablets plus olsalazine sodium capsules can reduce inflammatory factors in the treatment of ulcerative colitis patients. The mechanism may be due to the active probiotics of the bifidobacterium lactobacillus triple viable bacteria capsules [23], and the active probiotics can multiply in the intestines, increase the ratio of beneficial bacteria in the colorectal, inhibit the reproduction of pathogenic bacteria, and reduce the bacteria, improve the body's ability to absorb nutrients [24], thereby reducing the inflammatory response, and with the extension of time, the inflammatory response became increasingly lower [25].

The imbalance between the various subgroups of T cells will induce an immune response, as well as damage to the cells and aggravation of the inflammatory response [26]. The increase of CD8+ cells will enhance the function of CD4+ cells, promote activation of many B lymphocytes, activate humoral immunity, AND cause the production of a large number of immune complexes, thereby inducing mucosal congestion, edema, and ulcer formation [26]. The study by Shen Hao [26] confirmed that ulcerative colitis patients showed disorders of T cell subpopulations. Yu Haiping and colleagues [27] showed that the combination of bifid triple viable tablets plus mesalazine can improve the clinical efficacy and effectively improve the immune function of patients who were treated fro ulcerative colitis. The results of this study showed that the CD4+ and CD4/CD8 ratios of the experimental group were higher than those of the control group, and the CD8+ level was lower than that of the control group (*P* < 0.05), suggesting that mesalazine plus somatostatin and bifidus triple capsules can effectively regulate T cell frequency of ulcerative colitis patients. Lu Lei et al. [28] confirmed that the adjuvant treatment of ulcerative colitis with bifid triple viable bacteria can regulate the levels of oxidative stress and inflammatory factors, inhibit the body's inflammatory response, and improve immune function. Studies by Tang Xuejun [25] and colleagues have confirmed that the adjuvant treatment of ulcerative colitis with bid triple viable powder can help inhibit the body's inflammatory response and improve immune function, and the effect was time dependent. The results of this study showed that after treatment, the CD4+ and CD4/CD8 ratios of the experimental group were higher than those of the control group, and the CD8+ levels were lower than that of the control group (*P* < 0.05), suggesting that mesalazine plus somatostatin and bifid triple viable capsules can effectively regulate the immune function of ulcerative colitis patients. The mechanism may be due to the fact that the bifid triple viable capsule can increase the phagocytic ability of macrophages in the gastrointestinal tract, stimulate the body to secrete a large amount of IgA and IgG, prolong the lifespan of T lymphocytes, mediate phagocytosis, and maintain the balance of the intestinal flora, improve the immune function of the body, and with the extension of the time, the immune function will also increase [25].

The main pathological characteristics of patients with inflammatory bowel disease is the damage of intestinal mucosal barrier function [29], which is related to inflammatory response, mucosal immune function, intestinal mucosal permeability changes and intestinal mucosal membrane cell defects, which can accelerate intestinal mucosal cell apoptosis and increase intestinal endotoxins, or even induce intestinal failure [29]. Therefore, it is necessary to repair the intestinal mucosal barrier function of patients in the treatment of ulcerative colitis. Plasma D-lactic acid is a commonly used sensitive indicator that reflects the permeability of the intestine, and most of the intracellular enzymes exist in the small intestinal mucosa [30]. ET is an effective indicator of the damage of intestinal function and is composed of the cell wall of gram-negative bacteria [30]. DA0 is an effective indicator of the damage and repair of the intestinal barrier [30]. Most of the intracellular enzymes exist in the small intestinal mucosa. Zhao et al. [31] have confirmed that the plasma levels of D-lactic acid, ET, and DAO in ulcerative colitis patients were abnormally elevated and closely related to the severity of the disease. Feng Xianqing [32] confirmed that bifid triple viable capsules plus mesalazine can help improve the clinical efficacy of ulcerative colitis patients, which may be related to the improvement of immune function and the function of intestinal mucosal barrier of patients. The results of this study showed that the plasma D-lactic acid, ET and DAO of the experimental group were lower than those of the control group after treatment (*P* < 0.05), suggesting that mesalazine plus somatostatin and bifid triple viable capsules can effectively reduce the plasma D-lactic acid, ET, and DA0 levels of ulcerative colitis patients, thereby restoring the markers for intestinal mucosal barrier function. This may be because the bifid triple viable capsule can promote the repair and regeneration of intestinal epithelial tissue cells by producing butyric acid and acetic acid, forming a protective layer between the intestinal mucosa and microorganisms, and enhancing the barrier function of the intestinal mucosa [33].

Ulcerative colitis patients will be affected by psychology, society, and physiology during the development of the disease, which will affect the QoL of patients to varying degrees [34]. In recent years, with the transformation of the medical model to the physiological-psychological-social model, the QoL of patients has become a hot spot in clinical research, and it has also become one of the effective evaluation indicators after disease treatment [35]. Therefore, this study evaluated the QoL of ulcerative colitis patients after the treatment with mesalazine plus somatostatin and bifid triple viable capsules. The results showed that emotional ability, social ability, intestinal and systemic symptoms of patients in the experimental group were higher than those of the control group (*P* < 0.05), suggesting that mesalamine plus somatostatin and bifid triple viable capsules can effectively improve the QoL of ulcerative colitis patients. Wang Jia [36] confirmed that kangfuxin liquid plus bifid triple viable capsules can improve the QoL of ulcerative colitis patients, which is consistent with the results of this study. This study further analyzed the AEs and the results showed that the incidence of AEs in the two groups was comparable (*P* > 0.05), suggesting that mesalazine plus somatostatin and bifid triple viable capsules have relatively good safety and do not increase the AEs in ulcerative colitis patients.

### Limitations

This study has some limitations. The sample size is small, and it is a single-center randomized controlled study. In future studies, a multi-center randomized controlled study with a large sample size should be conducted to further confirm the conclusions of this study. In addition, there is no placebo for the bifid triple viable capsules, which might affect the conclusions of this study. We will conduct clinical trials with placebo to further confirm these conclusions.

## Conclusion

In conclusion, mesalazine plus somatostatin and bifid triple viable capsules can effectively improve the clinical symptoms of ulcerative colitis patients. It can reduce plasma inflammatory factors, regulate T cell frequency, restore markers for intestinal mucosal barrier function, and improve QoL, with high safety.

## Data Availability

The datasets generated and analyzed during the current study are available from the corresponding author on reasonable request.

## Abbreviations

ET: Endotoxin
DA0: Diamine oxidase
IL-6: Interleukin-6
IL-8: Interleukin-8
hs-CRP: High-sensitivity C-reactive protein
TNF-α: Tumor necrosis factor
